# Supplementary material for: Protection of Liver Functions and Improvement of Kidney Functions by Twelve Weeks Consumption of Cuban Policosanol (Raydel®) with a Decrease of Glycated Hemoglobin and Blood Pressure from a Randomized, Placebo-Controlled, and Double-Blinded Study with Healthy and Middle-Aged Japanese Participants
Source: Life (Basel). 2023 Jun 4;13(6):1319. doi: 10.3390/life13061319 (PMC10301011; doi:10.3390/life13061319)
Supplement: Supplementary file 1 [file life-13-01319-s001.zip › life-2372833-supplementary.pdf]

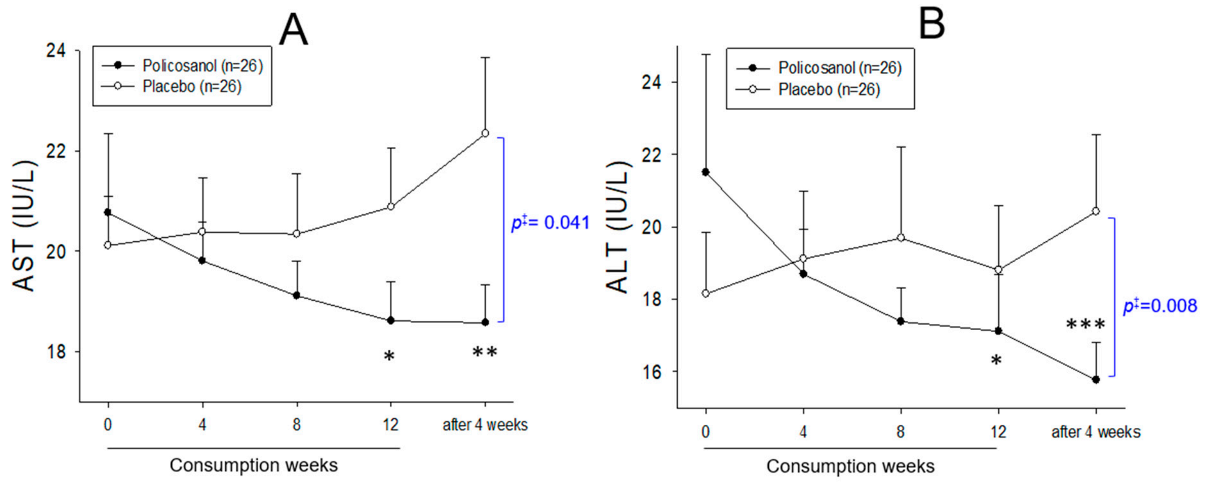

Supplemental Figure S1. Graphical expression of change in the parameters in hepatic function, serum AST (A), and ALT (B), during the 16 weeks between the policosanol and placebo group. AST, Aspartate transaminase; ALT, Alanine aminotransferase. *, $p<0.05$ versus placebo; **, $p<0.01$ versus placebo; ***, $p<0.001$ versus placebo from the analysis of covariance (ANCOVA) model with the independent variable as the baseline and treatment. p‡ value in blue font indicates the significance of time and group interaction during 16 weeks from repeated measurement ANOVA.

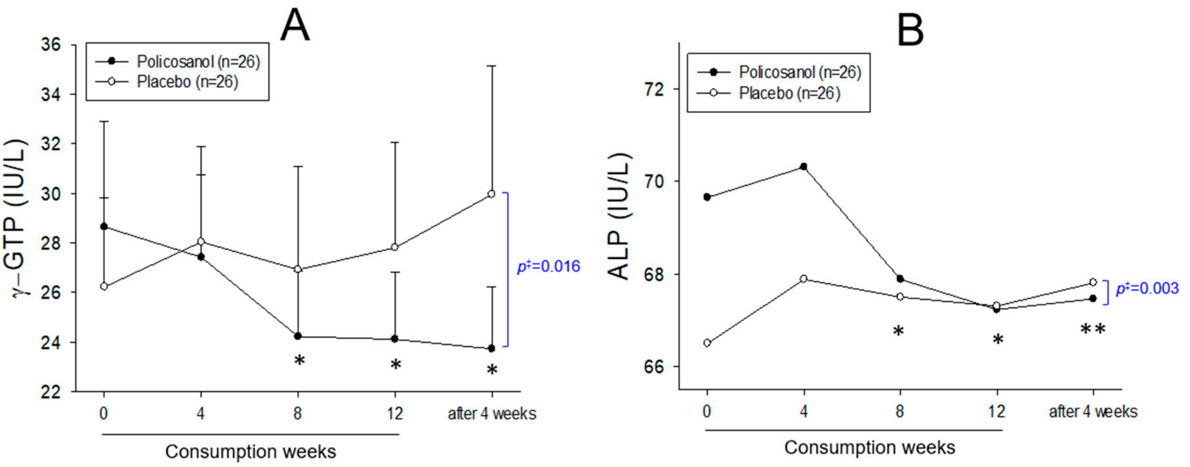

Supplemental Figure S2. Graphical expression of change in the parameters in hepatic function, serum γ-GTP (A) and ALP (B), during the 16 weeks between the policosanol and placebo group. γ-GTP, Gamma-glutamyl transferase; ALP, Alkaline phosphatase. *, $p<0.05$ versus placebo from the analysis of covariance (ANCOVA) model with the independent variable as the baseline and treatment. p‡ value in blue font indicates the significance of time and group interaction during 16 weeks from repeated measurement ANOVA.

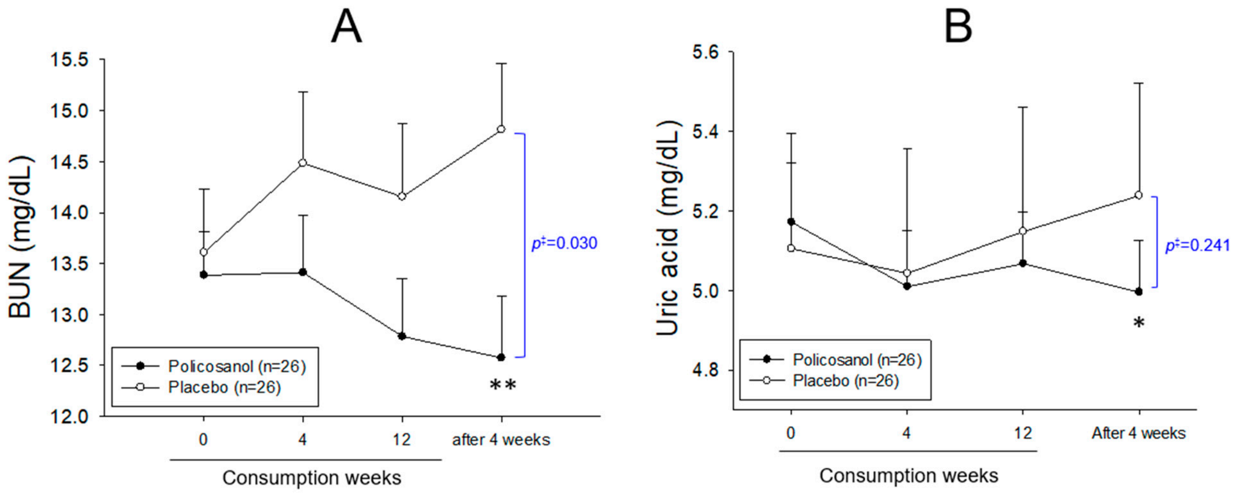

Supplemental Figure S3. Graphical expression of change in the parameters of the kidney functions, blood urea nitrogen (A), and uric acid (B), during the 16 weeks between the policosanol and placebo group. BUN, blood urea nitrogen. *, p<0.05 versus placebo; **, p<0.01 versus placebo from the analysis of covariance (ANCOVA) model with the independent variable as the baseline and treatment. p‡ value in blue font indicates the significance of time and group interaction during 16 weeks from repeated measurement ANOVA.

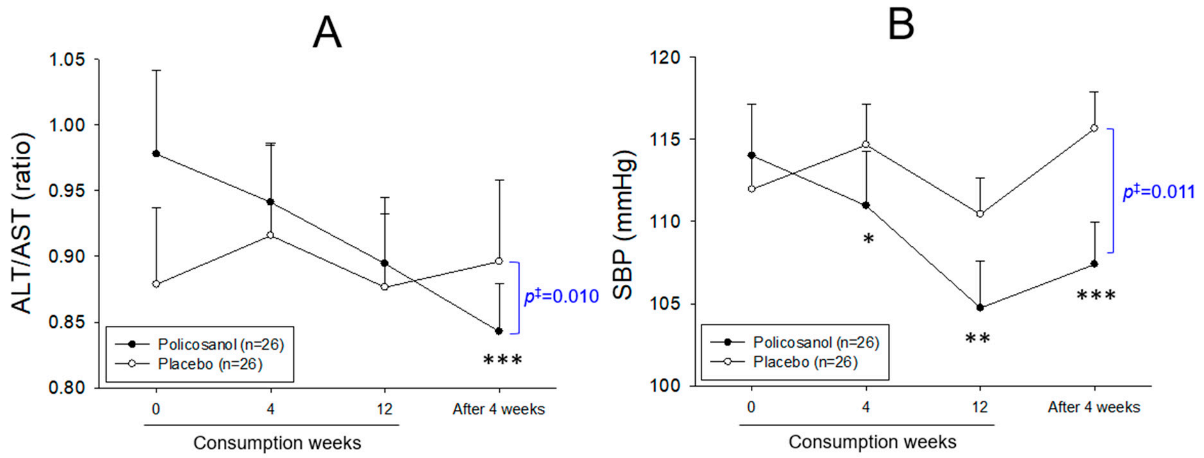

Supplemental Figure S4. Graphical expression of change in the hepatic parameters, ALT/AST ratio, (A) and SBP (B) during the 16 weeks between the policosanol and placebo group. *, p<0.05 versus placebo; **, p<0.01 versus placebo; ***, p<0.001 versus placebo from the analysis of covariance (ANCOVA) model with the independent variable as the baseline and treatment. p‡ value in blue font indicates the significance of time and group interaction during 16 weeks from repeated measurement ANOVA.

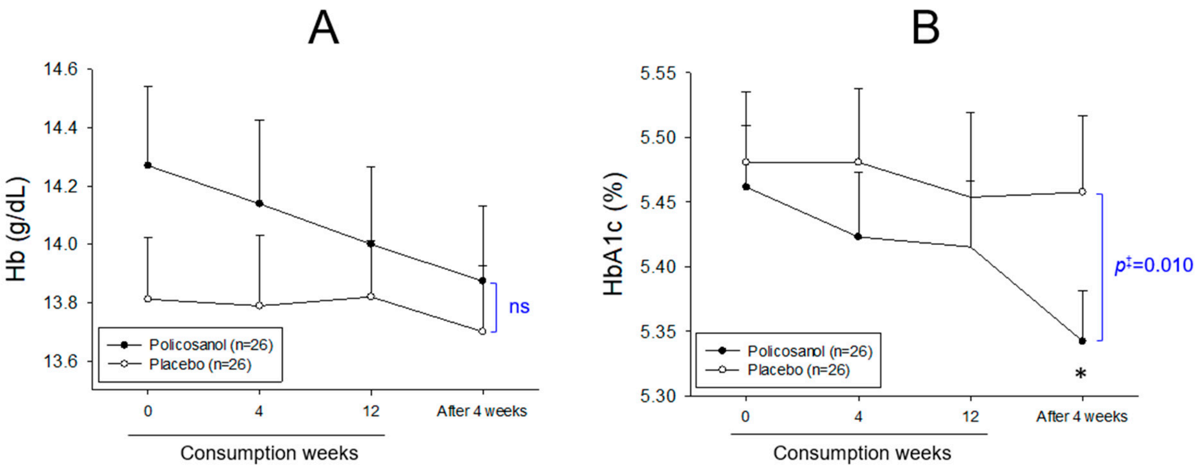

Supplemental Figure S5. Graphical expression of change in the blood hemoglobin (A) and glycated hemoglobin (B) contents during the 16 weeks between the policosanol and placebo groups. Hb, hemoglobin; HbA1c, glycated hemoglobin. *, $p<0.05$ versus the placebo from the analysis of covariance (ANCOVA) model with the independent variable as the baseline and treatment. $p^{‡}$ value in blue font indicates the significance of the time and group interaction during 16 weeks from repeated measurement ANOVA.

Supplemental Table S1. Comparison of baseline (week 0) data between placebo and policosanol 20 mg group. ¶

Variables	Placebo (n=26)	Policosanol 20 mg (n=26)	*p*	Normal range
SBP (mmHg)	112.0±2.1	114.0±3.1	0.586	< 120 mmHg
DBP (mmHg)	67.8±1.6	69.3±1.9	0.561	< 80 mmHg
pulse rate (BPM)	72.1±2.0	69.6±2.1	0.400	60 – 100 beat/min
Weight (kg)	60.5±1.9	58.8±1.8	0.520	
BMI (kg/㎡)	22.3±0.5	21.8±0.5	0.467	18.5 – 22.9
WBC (10^4/μL)	4.6±0.2	4.5±0.2	0.870	0.4 – 10
RBC (10^4/μL)	451.7±7.5	467.7±7.2	0.132	Male: 400 – 550<br>Female: 350 – 450
Plt (10^4/μL)	24.9±1.0	25.8±1.1	0.516	15 – 40
Hb (g/dL)	13.8±0.2	14.3±0.3	0.187	Male: 13.5 – 17.5<br>Female: 12.5 – 15.5
HbA1c (%)	5.48±0.05	5.46±0.05	0.791	< 6.0
Hct (%)	40.6±0.7	41.8±0.8	0.256	Male: 39 – 51<br>Female: 33 – 45
MCV (fl)	90.0±0.8	89.3±1.0	0.600	80 - 95
MCH (pg)	30.6±0.3	30.6±0.4	0.886	27.3 – 38
MCHC (%)	34.0±0.2	34.2±0.3	0.693	33 – 36
Total protein (g/dL)	7.0±0.1	7.1±0.1	0.150	6 – 8
Albumin (g/dL)	4.33±0.04	4.37±0.05	0.573	3.5 – 5.2
LDH (IU/L)	164.9±5.3	159.0±4.8	0.414	100 - 225
Creatinine (mg/dL)	0.77±0.03	0.72±0.02	0.225	0.7 – 1.4
Glucose (mg/dL)	90.2±1.8	89.5±1.8	0.314	70 - 99
hsCRP (mg/dL)	0.12±0.08	0.05±0.02	0.346	0.00 – 0.49
AST (IU/L)	20.1±1.0	20.8±1.6	0.724	5 – 40
ALT (IU/L)	18.2±1.7	21.5±3.3	0.367	0 – 40
ALT/AST (ratio)	0.88±0.06	0.98±0.06	0.258	
γ-GTP (IU/L)	26.2±3.6	28.7±4.3	0.666	Male: 11 - 63<br>Female: 8 – 35
ALP (IU/L)	64.8±3.9	69.0±2.6	0.414	30 - 115
BUN (mg/dL)	13.6±0.6	13.4±0.4	0.767	10 - 26
Uric acid (mg/dL)	5.1±0.3	5.2±0.2	0.859	Male: 3 – 7<br>Female: 2.5 – 5.5

¶ Data were expressed as mean ± SEM. Significant differences were analyzed between groups with t-test using the SPSS software version 29.0 (IBM, Chicago, IL, USA).

AST, alanine transaminase; ALT, alanine aminotransferase; γ-GTP, gamma-glutamyl transferase; ALP, alkaline phosphatase; HbA1c, glycated hemoglobin; BUN, blood urea nitrogen; DBP, diastolic blood pressure. BMI, body mass index; BPM, beat per minute; BUN, blood urea nitrogen; SBP, systolic blood pressure; WBC, White blood cell; RBC, red blood cell; Hb, hemoglobin; HbA_{1c}, glycated hemoglobin; Hct, hematocrit; MCV, mean corpuscular volume; MHC, mean corpuscular hemoglobin; MCHC, mean corpuscular hemoglobin concentration; Plt, platelets; hsCRP, high-sensitivity C-reactive protein; LDH, lactate dehydrogenase

Supplemental Table S2. Parameters for kidney function test

Variables	Placebo (n=26) PCO 20mg (n=26)	Week 0	Week 4	Week 8	Week12	post 4 weeks
		Mean ± SEM	Mean ± SEM	Mean ± SEM	Mean ± SEM	Mean ± SEM
P (mg/dL)	**placebo**	3.52±0.06	3.67±0.10	3.55±0.10	3.51±0.08	3.63±0.08
	**PCO 20 mg**	3.46±0.08	3.51±0.07	3.40±0.09	3.43±0.08	3.45±0.09
	p	0.534	0.260	0.336	0.714	0.139
Ca (mg/dL)	**placebo**	9.32±0.06	9.39±0.07	9.22±0.07	9.26±0.07	9.23±0.06
	**PCO 20 mg**	9.37±0.05	9.32±0.06	9.20±0.05	9.24±0.05	9.18±0.05
	p	0.517	0.169	0.404	0.459	0.114
Na (mg/dL)	**placebo**	140.9±0.2	140.7±0.2	140.7±0.3	141.3±0.2	141.2±0.3
	**PCO 20 mg**	140.4±0.3	140.7±0.3	140.5±0.4	141.3±0.2	140.7±0.3
	p	0.202	0.452	0.706	0.535	0.767
K (mg/dL)	**placebo**	4.13±0.06	4.20±0.07	4.1±0.05	4.12±0.05	4.07±0.05
	**PCO 20 mg**	4.22±0.04	4.11±0.06*	4.1±0.06	4.17±0.04	4.07±0.05
	p	0.200	0.043	0.418	0.739	0.381
Cl (mg/dL)	**placebo**	105.3±0.3	104.0±0.4	103.9±0.3	105.2±0.3	105.1±0.3
	**PCO 20 mg**	104.5±0.3	103.9±0.3	103.7±0.4	104.4±0.3	104.6±0.3
	p	0.036	0.43	0.536	0.463	0.902

Data are expressed as mean±SEM, *, p<0.05 vs week 0 in each group. P, inorganic phosphorus; Ca, calcium; Na, sodium; K, potassium; Cl, chloride.

Supplemental Table S3. Change in the blood pressure and anthropological data between placebo group and policosanol (PCO) group during 16 weeks.[¶]

Variables	Placebo (n=26) / PCO 20 mg (n=26)	Week 0 Mean ± SEM	Week 4 Mean ± SEM	Week 8 Mean ± SEM	Week 12 Mean ± SEM	post 4 weeks Mean ± SEM
SBP (mmHg)	placebo	112.0±2.1	114.7±2.5	105.9±3.1	110.4±2.2	115.7±2.2
	PCO 20 mg	114.0±3.1	111.0±3.3	104.0±2.8	104.7±2.9	107.4±2.6*
	$p^{\dagger}$	0.586	0.045	0.231	0.004	0.001
DBP (mmHg)	placebo	67.8±1.6	66.1±1.7	63.4±1.8	67.9±1.4	69.1±1.6
	PCO 20 mg	69.3±1.9	67.0±2.1	62.7±1.9	66.9±2.3	67.3±1.8
	$p^{\dagger}$	0.561	0.868	0.268	0.255	0.171
Pulse rate (BPM)	placebo	72.1±2.0	72.1±1.9	71.9±2.2	72.0±2.5	71.8±1.8
	PCO 20 mg	69.6±2.1	70.0±2.2	68.8±1.8	68.8±2.0	70.9±1.8
	$p^{\dagger}$	0.400	0.836	0.472	0.607	0.835
Body weight (kg)	placebo	60.5±1.9	60.3±1.9	60.5±1.9	60.1±1.8	60.3±1.8
	PCO 20 mg	58.8±1.8	59.1±1.9	59.0±1.9	58.6±1.8	58.5±1.8
	$p^{\dagger}$	0.520	0.031	0.361	0.610	0.698
BMI (kg/㎡)	placebo	22.3±0.5	22.2±0.5	22.2±0.5	22.1±0.5	22.2±0.5
	PCO 20 mg	21.8±0.5	21.9±0.5	21.8±0.5	21.7±0.5	21.7±0.5
	$p^{\dagger}$	0.467	0.022	0.316	0.633	0.659

[¶] Data are expressed as mean ± SEM. The estimated statistical power is 99.8% based on calculations using G*Power 3.1.9.7 (G*Power from the University of Düsseldorf, Düsseldorf, Germany). *, $p<0.05$; **, $p<0.01$ from a paired *t*-test obtained between the two groups at each time point. $p^{\dagger}$, from analysis of covariance (ANCOVA) model with the independent variable as baseline and treatment vs. week 0 in each group. BMI, body mass index; BPM, beat per minute; DBP, diastolic blood pressure; PCO, policosanol; SBP, systolic blood pressure.

Supplemental Table S4. Changes in the hematologic data in blood between placebo group and policosanol (PCO) group during 16 weeks.¶

Variables	Placebo (n=26) / PCO 20mg (n=26)	Week 0 Mean ± SEM	Week 4 Mean ± SEM	Week 8 Mean ± SEM	Week12 Mean ± SEM	post 4 weeks Mean ± SEM
WBC (10^4/μL)	placebo	4.6±0.2	4.5±0.2	4.6±0.2	4.7±0.2	4.8±0.2
	PCO 20 mg	4.5±0.2	4.7±0.3	4.9±0.2	4.7±0.2	4.8±0.2
	$p^{\dagger}$	0.870	0.463	0.194	0.700	0.858
RBC (10^4/μL)	placebo	451.7±7.5	446.8±8.0	443.8±7.3	446.0±7.1	444.1±8.1
	PCO 20 mg	467.7±7.2	460.2±7.5	455.0±7.7	455.3±7.2	450.2±6.7*
	$p^{\dagger}$	0.132	0.602	0.447	0.282	0.043
Plt (10^4/uL)	placebo	24.9±1.0	25.1±1.0	23.4±0.9	24.7±1.1	24.3±0.9
	PCO 20 mg	25.8±1.1	25±0.9	24.1±0.8	24.9±0.9	24.8±0.9
	$p^{\dagger}$	0.516	0.134	0.925	0.457	0.616
Hb (g/dL)	placebo	13.8±0.2	13.8±0.2	13.9±0.2	13.8±0.2	13.7±0.2
	PCO 20 mg	14.3±0.3	14.1±0.3	14.2±0.3	14.0±0.3	13.9±0.3
	p	0.187	0.379	0.315	0.144	0.07
HbA_{1c} (%)	placebo	5.48±0.05	5.48±0.06	5.35±0.05	5.45±0.07	5.46±0.06
	PCO 20 mg	5.46±0.05	5.42±0.05	5.30±0.04	5.42±0.05	5.34±0.04*
	$p^{\dagger}$	0.791	0.230	0.250	0.599	0.004
Hct (%)	placebo	40.6±0.7	40.8±0.7	40.2±0.5	40.4±0.5	40.1±0.6
	PCO 20 mg	41.8±0.8	41.8±0.8	40.9±0.7	41.0±0.7	40.4±0.6
	$p^{\dagger}$	0.256	0.961	0.723	0.555	0.160
MCV (fL)	placebo	90.0±0.8	91.4±0.7	90.9±0.8	90.7±0.8	90.7±0.9
	PCO 20 mg	89.3±1.0	90.8±10	90.1±1.0	90.2±1.0	89.7±1.0
	$p^{\dagger}$	0.600	0.963	0.764	0.969	0.665
MCH (pg)	placebo	30.6±0.3	30.9±0.3	31.4±0.3	31±0.3	30.9±0.3
	PCO 20 mg	30.6±0.4	30.7±0.4	31.2±0.4	30.8±0.4	30.8±0.4
	$p^{\dagger}$	0.886	0.600	0.450	0.110	0.976
MCHC (%)	placebo	34.0±0.2	33.8±0.1	34.5±0.1	34.2±0.1	34.1±0.2
	PCO 20 mg	34.2±0.3	33.8±0.1	34.6±0.2	34.2±0.2	34.3±0.2
	$p^{\dagger}$	0.693	0.973	0.854	0.600	0.358

¶ Data are expressed as mean±SEM. Estimated statistical power is 99.8% based on the calculation using the program G*Power 3.1.9.7 (G*Power from the University of Düsseldorf, Düsseldorf, Germany).

*, $p<0.05$; **, $p<0.01$ from paired t-test obtained between the two groups in each time point. $p^{\dagger}$, from an analysis of covariance (ANCOVA) model with the independent variable as the baseline and treatment versus week 0 in each group. WBC, white blood cell; RBC, red blood cell; Hb, hemoglobin; HbA_{1c}, glycated hemoglobin; Hct, hematocrit; MCV, mean corpuscular volume; MHC, mean corpuscular hemoglobin; MCHC, mean corpuscular hemoglobin concentration; Plt, platelets.

Supplemental Table S5. Serum proteins, glucose, and inflammatory parameters between placebo group and policosanol (PCO) group during 16 weeks.¶

Variables	Placebo (n=26)<br><br>PCO 20mg (n=26)	Week 0	Week 4	Week 8	Week 12	post 4 weeks
		Mean ± SEM	Mean ± SEM	Mean ± SEM	Mean ± SEM	Mean ± SEM
Total protein (g/dL)	placebo	7.0±0.1	7.0±0.1	7.0±0.1	7.0±0.1	7.0±0.1
	PCO 20 mg	7.1±0.1	7.0±0.1	7.0±0.1	7.1±0.1	7.0±0.1
	$p^{\dagger}$	0.150	0.112	0.276	0.316	0.410
Albumin (g/dL)	placebo	4.33±0.04	4.40±0.05	4.31±0.04	4.35±0.05	4.34±0.05
	PCO 20 mg	4.37±0.05	4.35±0.05	4.30±0.05	4.35±0.05	4.34±0.06
	$p^{\dagger}$	0.573	0.160	0.458	0.554	0.423
LDH (IU/L)	placebo	164.9±5.3	166.0±5.8	164.2±5.0	165.5±5.2	168.3±6.2
	PCO 20 mg	159.0±4.8	155.6±4.5	155.9±4.7	158.5±4.4	155.6±4.8
	$p^{\dagger}$	0.414	0.118	0.264	0.525	0.111
Creatinine (mg/dL)	placebo	0.77±0.03	0.77±0.03	0.76±0.03	0.76±0.04	0.77±0.04
	PCO 20 mg	0.72±0.02	0.73±0.02	0.71±0.02	0.72±0.02	0.73±0.02
	$p^{\dagger}$	0.225	0.424	0.817	0.974	0.380
Glucose (mg/dL)	placebo	9.02±1.8	90.0±1.4	92.3±1.8	92.6±1.7	92.7±2.0
	PCO 20 mg	89.5±1.8	89.0±1.6	90.0±1.7	90.0±1.5	90.3±1.3
	$p^{\dagger}$	0.314	0.585	0.763	0.589	0.692
hsCRP (mg/dL)	placebo	0.12±0.08	0.22±0.16	0.04±0.01	0.04±0.01	0.11±0.05
	PCO 20 mg	0.05±0.02	0.05±0.01	0.05±0.01	0.03±0	0.03±0.01
	$p^{\dagger}$	0.346	0.293	0.433	0.410	0.151

¶ Data are expressed as mean±SEM. Estimated statistical power is 99.8% based on the calculation using the program G*Power 3.1.9.7 (G*Power from the University of Düsseldorf, Düsseldorf, Germany). *, $p<0.05$; **, $p<0.01$ from paired t-test obtained between the two groups in each time point. $p^{\dagger}$, from an analysis of covariance (ANCOVA) model with the independent variable as the baseline and treatment versus week 0 in each group. hsCRP, high-sensitivity C-reactive protein; LDH, lactate dehydrogenase; PCO, policosanol.
